# The comparative effectiveness of traditional Chinese medicine exercise therapies in elderly people with mild cognitive impairment: A protocol for a network meta-analysis

**DOI:** 10.1097/MD.0000000000022021

**Published:** 2020-09-04

**Authors:** Kai-Qi Su, Su-Tong Liu, Jie Yuan, Jie-Ying Li, Rui-Qing Li, Xiao-Dong Feng

**Affiliations:** a Henan University of Chinese Medicine; b Rehabilitation Center, The First Affiliated Hospital of Henan University of Chinese Medicine, Zhengzhou, Henan, PR China.

**Keywords:** Baduan jin exercise, exercise therapy, finger exercise, Liuzi jue, mild cognitive impairment, network meta-analysis, Tai Ji

## Abstract

**Background:**

Mild cognitive impairment (MCI) in the elderly is a health problem worldwide. Several clinical trials indicated that traditional Chinese medicine (TCM) exercise therapies can effectively improve MCI, such as Tai Ji, Baduan jin exercise, Liuzi jue, and finger exercise. However, there is still controversy over which therapy is the best for elderly MCI patients. In this study, we aimed to systematically evaluate and compare the effectiveness and safety of these 4 TCM exercise therapies in elderly patients with MCI.

**Methods:**

The Web of Science, PubMed, EMBASE, Cochrane Central Register of Controlled Trials, Clinical Trials, China National Knowledge Infrastructure, Wangfang database, and Chinese Biomedical Medicine will be comprehensively searched to collect all randomized controlled trials which included elderly participants with MCI receiving TCM exercise therapies through July 2020. Two reviewers will independently screen and evaluate each included study and extract the outcome indexes. ADDIS 1.16.8 software will be used for the network meta-analysis and STATA 14 software will be used for drawing network evidence plots and funnel plots.

**Results:**

We will use the Bayesian statistical model to conduct a network meta-analysis to rank the effectiveness and safety of these 4 interventions, and use the GRADE approach to interpret the results.

**Conclusion:**

This network meta-analysis will find out the optimal treatment plan for MCI and provide evidence-based bias for clinical treatments decision-making.

**Protocol registration number:**

INPLASY202070006.

## 1 Introduction

Mild cognitive impairment (MCI) in the elderly is one of the common functional disorders that mainly manifests as a decline in memory and execution, which is defined as the transitional stage of dementia and seriously affects the patient's overall recovery.^[1–3]^ Studies have shown that about 80% of MCI patients will progress to dementia within 5 years without timely intervention and treatment.^[4,5]^ In addition, it is estimated that the number of dementia patients worldwide will reach 115 million by 2050, which will bring a great burden to families and society.^[6]^

Early intervention can effectively delay or even prevent the progression of MCI to dementia.^[7,8]^ At present, MCI's drug interventions mainly include symptomatic treatments (such as cholinesterase inhibitors, N-methyl-D-aspartic acid receptor, ergot alkaloid preparations, etc) and etiological treatments (such as antidiabetic drugs, antihypertensive drugs, etc).^[9,10]^ However, certain side effects and adverse reactions do exist in drug interventions, so the current clinical treatments are still based on non-drug interventions.^[11–15]^ As an important component of non-drug intervention, traditional Chinese medicine (TCM) exercise therapies have a good curative effect on the body and mood by combining the holistic view of TCM and ancient philosophical concepts, and have been confirmed by several studies to improve cognitive function and widely used in China in recent years, such as Tai Ji, Baduan jin exercise, Liuzi jue, and finger exercise.^[16–19]^ However, the form and duration of these therapies are various. There is no strong evidence to support which therapy is appropriate for MCI patients.

Thus, this study is aimed to systematically evaluate the efficacy and safety of TCM exercise therapies for the treatment of MCI in the elderly. This network meta-analysis will be used to indirectly compare the 4 therapies to find out the optimal treatment plan for MCI and to provide evidence-based bias for clinical treatments decision-making.

## 2 Methods

### 2.1 Design and registration

This study will be reported in accordance with the guidelines of Cochrane Handbook for Systematic Reviews of Interventions and the Preferred Reporting Items for Systematic Review and Meta-Analyses Statement.^[20]^ This protocol has been registered on the INPLASY website (https://inplasy.com/inplasy-2020-7-0006/) and the registration number is INPLASY202070006. No ethical approval is required since this study used data already in the public domain.

### 2.2 Inclusion criteria

#### 2.2.1 Type of studies

Study type to be included in this review will be clinical randomized controlled trials (RCTs) that have been published or registered but not yet published in both Chinese and English. Animal experiments, case reports, reviews, and non-RCTs will be excluded.

#### 2.2.2 Type of participants

Participants to be included in this review will be elderly people (over 60 years old) who are diagnosed with MCI using mental state examinations, and sufficient memory and hearing will be necessary to receive exercise therapies. There will be no restriction on gender, region, or race. Participants who cannot be clearly diagnosed as MCI and participants with severe heart disease, liver and kidney dysfunction, mental illness will be excluded.

#### 2.2.3 Type of interventions

Four types of TCM exercise therapies will be included in this study, including Tai Ji, Baduan jin exercise, Liuzi jue, and finger exercise. All 4 therapies listed above can be used as monotherapy or combined treatments in the intervention group, and the controlled interventions will be routine nursing intervention or other therapies mentioned above that are different from the intervention group. If there are uncertain interventions, this study will be excluded.

#### 2.2.4 Outcome assessments

The primary outcomes include the Montreal cognition assessment scale and the Mini-mental state examination scale.

The secondary outcomes include the activity of daily living scale, Trail Making Test, adverse events, and so on.

### 2.3 Data sources and searches

We will search publications in the following English and Chinese databases: Web of Science, PubMed, EMBASE, Cochrane Central Register of Controlled Trials, Clinical Trials, China National Knowledge Infrastructure, Wangfang database, and Chinese Biomedical Medicine. The search strategy will include medical subject headings and free words associated with TCM exercise therapies in the treatment of MCI in the elderly. Subject headings include “exercise therapy,” “Tai Ji,” “Baduan jin exercise,” “Liuzi jue,” “finger exercise,” “mild cognitive impairment,” and so on. The retrieval time will be set to the time of database-building to July, 2020. In Table 1, we present the search strategy of the Cochrane Library.

**Table 1:**
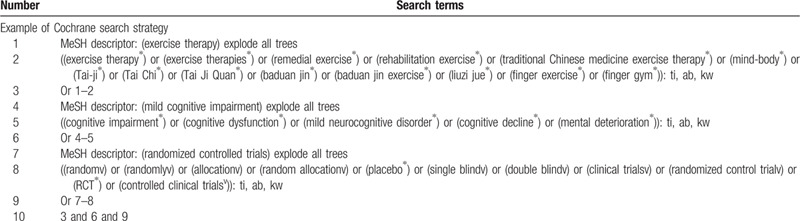
Cochrane library search strategy.

### 2.4 Data collection and analysis

#### 2.4.1 Selection of studies

For the convenience of management, we will import all the retrieved literature data that searched from above-mentioned databases into Endnote X7 software. Two independent researchers will screen titles and abstracts to identify potentially eligible studies, and remove studies that obviously do not meet the inclusion criteria. Then, we will download the full text of the remaining studies, read them carefully and decide which ones to include, and record the reasons for exclusion. Finally, the 2 researchers will cross-check the selection results. If any disagreements were found, we will reach agreement through discussion or seek advice from a third researcher.

#### 2.4.2 Data extraction

The 2 researchers will independently extract the data using Excel 2019 software. The contents of extracted data include the name of the first author, the time of publication, country, sample size, characteristics of participants, intervention details, duration, outcome measures, and so on. Similarly, the results of data extraction will be cross-check by the 2 researchers, and any diversities will be resolved through discussion or a third researcher.

#### 2.4.3 Assessment of risk of bias

The 2 researchers will use the RCT bias risk assessment tool recommended by the Cochrane Handbook for Systematic Reviews of Interventions 5.1.0 to perform bias risk assessment and methodological quality assessment of included RCTs.^[21,22]^ Any disagreement will be handled by a third researcher.

#### 2.4.4 Data analysis

The ADDIS 1.16.8 software and the Bayesian hierarchical model will be used for this network meta-analysis. The odds ratio will be used as an effect indicator for dichotomous variables, and the weighted mean difference or standard mean difference will be used for continuous variables. The estimated value and 95% confidence interval will be used to represent the effect analysis statistics, and the significance level will be set at *α* = 0.05. Heterogeneity testing will be implemented to detect statistical heterogeneity between each study. If heterogeneity does not exist, then network meta-analysis will be performed directly; otherwise, we will analyze and describe the source of the heterogeneity, or only perform a descriptive analysis when the heterogeneity cannot be found. In addition, the node-split model will be used for the inconsistency test. If *P* > .05, it indicates that there is no significant difference between direct and indirect comparison, and the consistency model will be conducted for analysis; otherwise, the inconsistency model will be conducted.^[23,24]^ Simultaneously, a sorted probability table will be used to further analyze the advantages and disadvantages of therapies. The STATA 14 software will be used to draw a network diagram to visually present the comparisons between all therapies, and a funnel plot to make a qualitative judgment of publication deviation.

#### 2.4.5 Subgroup analysis

Subgroup analysis will be conducted with gender, race, nationality, duration of intervention, and the different causes of MCI to explore whether therapies effects for primary outcomes are robust.

#### 2.4.6 Assessment of publication bias

Funnel plots will be generated to evaluate the publication bias. If the funnel plots are symmetrically distributed, it means that there is no publication bias; otherwise, we will analyze and describe the possible reasons of asymmetry.^[25]^

#### 2.4.7 Assessment of quality of evidence

The grading of recommendations assessment, development, and evaluation system will be used to evaluate the quality of evidence. According to this system, the level of evidence quality will be divided into 4 levels: high, medium, low, and very low.^[26]^

## 3 Discussions

Cognitive impairment is a global burdensome problem in the elderly, which seriously affects the daily life and overall rehabilitation of patients. The complicated pathogenesis of cognitive impairment has not been studied clearly, and there is no approved intervention with specific efficacy and safety that is recommended for the treatment of cognitive impairment patients. Fortunately, exercise therapy is an alternative therapy for patients with MCI, and it has been widely promoted in most countries. In 2017, the practice guidelines for MCI formulated by the American Academy of Neurology recommended that MCI patients should be encouraged to exercise twice a week.^[27]^ Also in China, the 2030 healthy China plan also clearly stated that it is necessary to fully explore and play the role of TCM exercise therapies in the treatment of chronic diseases.

Therefore, this study will conduct a systematic review and network meta-analysis of related RCTs, and will comprehensively evaluate whether TCM exercise therapies are beneficial to elderly MCI patients. In addition, this study will compare the advantages and disadvantages of these 4 therapies and provide higher-quality evidence for the clinical treatment of MCI.

## Author contributions

**Conceptualization:** Kai-Qi Su, Su-Tong Liu.

**Data curation:** Kai-Qi Su, Su-Tong Liu, Jie Yuan, Jie-Ying Li.

**Formal analysis:** Kai-Qi Su, Rui-Qing Li.

**Funding acquisition:** Xiao-Dong Feng.

**Methodology**: Kai-Qi Su, Su-Tong Liu, Jie Yuan.

**Project administration:** Rui-Qing Li.

**Resources:** Kai-Qi Su, Su-Tong Liu, Jie Yuan.

**Software:** Kai-Qi Su, Su-Tong Liu.

**Supervision:** Xiao-Dong Feng.

**Writing – original draft:** Kai-Qi Su, Su-Tong Liu.

**Writing – review & editing:** Kai-Qi Su, Xiao-Dong Feng.
